# P-2258. Characterizing real world infection risk in multiple myeloma patients receiving teclistamab

**DOI:** 10.1093/ofid/ofae631.2411

**Published:** 2025-01-29

**Authors:** Emma Guare, Chen Song, Yoshitaka Inoue, Manpreet Sandhu, Jeffrey Sivik, Kevin Rakszawski, Seema Naik, Kentaro Minagawa, Shin Mineishi, Catharine I Paules

**Affiliations:** Penn State College of Medicine, Hershey, Pennsylvania; Penn State Health Milton S. Hershey Medical Center, Hershey, Pennsylvania; Penn State Cancer Institute, Hershey, Pennsylvania; Penn State Cancer Institute, Hershey, Pennsylvania; Penn State Health Milton S. Hershey Medical Center, Hershey, Pennsylvania; Penn State Cancer Institute, Hershey, Pennsylvania; Penn State Cancer Institute, Hershey, Pennsylvania; Penn State Cancer Institute, Hershey, Pennsylvania; Penn State Cancer Institute, Hershey, Pennsylvania; Penn State Health Milton S. Hershey Medical Center, Hershey, Pennsylvania

## Abstract

**Background:**

Teclistamab is an anti-B-cell maturation antigen (BCMA) bispecific antibody used in relapsed, refractory multiple myeloma (MM) that induces durable responses but is associated with high rates of infectious complications. Real world data are needed to further characterize infection risk in patients receiving teclistamab.

**Table 1.**
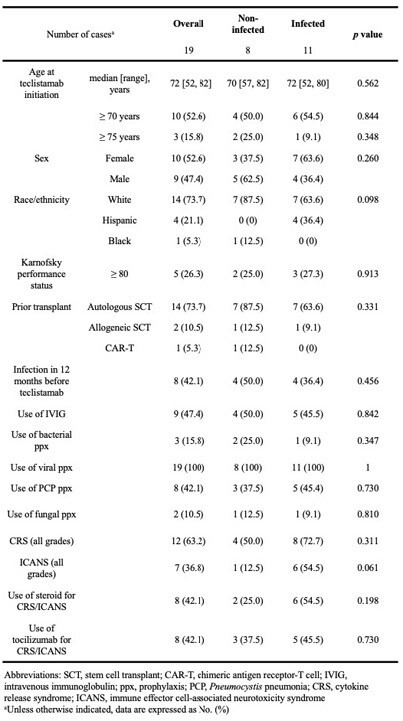
Patient characteristics between infected and non-infected patients

**Methods:**

A retrospective single-center study of teclistamab treated patients with relapsed, refractory MM from 1/1/2023 to 11/20/2023. Patients received teclistamab according to a step-up dosing regimen, with Day 0 defined as first step-up dose. The primary objective was to report the incidence, type, and timing of infection. Differences in demographics and clinical characteristics were described between infected and non-infected patients.

**Table 2.**
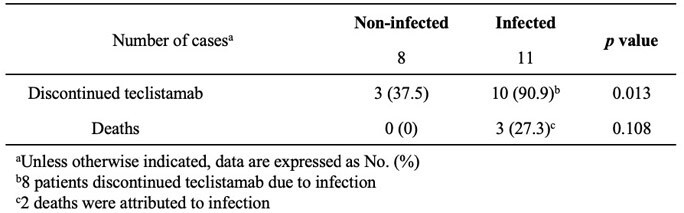
Patient outcomes on teclistamab between infected and non-infected patients

**Results:**

19 patients received teclistamab; median age 72 years, 73.7% white, and 26.3% with Karnofsky performance status ≥ 80 (Table 1). 11 (57.9%) patients developed infections and 5 had more than one infection. There were 3 bacteremias, 6 other bacterial infections (urinary tract infection, pneumonia, cholangitis, osteomyelitis, sinusitis), 6 respiratory viral infections, and 2 cytomegalovirus (CMV) reactivations. There were no documented infections with herpes simplex virus, varicella zoster virus, *Pneumocystis jirovecii,* or fungal pathogens. The median time to any infection was 17 days (range 2, 266), with a median of 20 days (range 2, 123) to bacterial infection and 31 days (range 3, 266) to viral infection (Figure 1). Patient characteristics and antimicrobial prophylaxis strategies were largely similar between infected and non-infected patients (Table 1). There was a nonsignificant trend toward increased immune effector cell-associated neurotoxicity syndrome (ICANS) and increased use of steroids in the infected group. 10 patients in the infected group discontinued teclistamab (p=0.013) and 3 died (p=0.108, Table 2).

**Figure 1.**
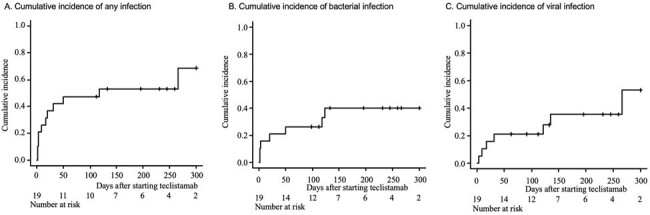
Cumulative incidence of any infection (A), bacterial infection (B), and viral infection (C).

**Conclusion:**

The incidence of infection was high amongst patients receiving teclistamab and increased with time. Individuals with infections were more likely to discontinue teclistamab. Further data are needed to inform guidance regarding infection prevention strategies and criteria for teclistamab interruption or discontinuation in this MM population with few other treatment options.

**Disclosures:**

Kevin Rakszawski, MD, AstraZeneca: Speakers Bureau|Pfizer: Speakers Bureau Seema Naik, MD, ADC Therapeutics: Advisor/Consultant|ADC Therapeutics: Board Member|ADC Therapeutics: Honoraria|Janssen: Advisor/Consultant|Janssen: Board Member|Janssen: Honoraria|Onclive: Honoraria|Onclive: Speaker

